# A Small *Wolbachia* Protein Directly Represses Phage Lytic Cycle Genes in “*Candidatus* Liberibacter asiaticus” within Psyllids

**DOI:** 10.1128/mSphereDirect.00171-17

**Published:** 2017-06-07

**Authors:** Mukesh Jain, Laura A. Fleites, Dean W. Gabriel

**Affiliations:** Department of Plant Pathology, University of Florida, Gainesville, Florida, USA; University of Wisconsin—Madison; University of California, Berkeley; USDA-ARS

**Keywords:** Huanglongbing, *Wolbachia*, citrus greening, cross talk, holin, phage repressor, psyllid, quorum sensing

## Abstract

Huanglongbing (HLB) is a severe disease of citrus caused by an uncultured alphaproteobacterium “*Candidatus* Liberibacter asiaticus” and transmitted by Asian citrus psyllids (*Diaphorina citri*). Two prophage genomes, SC1 and SC2, integrated in “*Ca*. Liberibacter asiaticus” strain UF506 were described previously, and very similar prophages are found resident in the majority of “*Ca*. Liberibacter asiaticus” strains described worldwide. The SC1 lytic cycle is marked by upregulation of prophage late genes, including a functional holin (SC1_gp110); these late genes are activated when “*Ca*. Liberibacter asiaticus” is *in planta*, but not when infecting the psyllid host. We previously reported that the holin promoter is strongly and constitutively active in Liberibacter crescens (a cultured proxy for uncultured “*Ca*. Liberibacter asiaticus”) but is suppressed in a dose-dependent manner by crude aqueous extracts from *D. citri* applied exogenously. Here we report that the suppressor activity of the crude psyllid extract was heat labile and abolished by proteinase K treatment, indicating a proteinaceous repressor and of a size smaller than 30 kDa. The repressor was affinity captured from *D. citri* aqueous extracts using biotinylated holin promoter DNA immobilized on magnetic beads and subjected to liquid chromatography-tandem mass spectrometry (LC-MS/MS). Protein database interrogation was used to identify a small DNA-binding protein encoded by a gene carried by *Wolbachia* strain wDi, a resident endosymbiont of *D. citri* as the repressor. The *in vitro*-translated *Wolbachia* repressor protein was able to penetrate *L*. *crescens* cells, bind to “*Ca*. Liberibacter asiaticus” promoter DNA, and partially suppress holin promoter-driven β-glucuronidase (GUS) activity, indicating potential involvement of an additional interacting partner(s) or posttranslational modification(s) for complete suppression. Expression of the *Wolbachia* repressor protein appeared to be constitutive irrespective of “*Ca*. Liberibacter asiaticus” infection status of the insect host.

**IMPORTANCE** Host acquisition of a new microbial species can readily perturb the dynamics of preexisting microbial associations. Molecular cross talk between microbial associates may be necessary for efficient resource allocation and enhanced survival. Classic examples involve quorum sensing (QS), which detects population densities and is both used and coopted to control expression of bacterial genes, including host adaptation factors. We report that a 56-amino-acid repressor protein made by the resident psyllid endosymbiont *Wolbachia* can enter cells of Liberibacter crescens, a cultured proxy for the uncultured psyllid endosymbiont “*Ca*. Liberibacter asiaticus” and repress “*Ca*. Liberibacter asiaticus” phage lytic cycle genes. Such repression in “*Ca*. Liberibacter asiaticus” may be critical to survival of both endosymbionts, since phage-mediated lysis would likely breach the immunogenic threshold of the psyllid, invoking a systemic and nonspecific innate immune reaction.

## Abstract (precis)

Host acquisition of a new microbial species can readily perturb the dynamics of preexisting microbial associations. Molecular cross talk between microbial associates may be necessary for efficient resource allocation and enhanced survival. Classic examples involve quorum sensing (QS), which detects population densities and is both used and coopted to control expression of bacterial genes, including host adaptation factors. We report that a 56-amino-acid repressor protein made by the resident psyllid endosymbiont *Wolbachia* can enter cells of Liberibacter crescens, a cultured proxy for the uncultured psyllid endosymbiont “*Ca*. Liberibacter asiaticus” and repress “*Ca*. Liberibacter asiaticus” phage lytic cycle genes. Such repression in “*Ca*. Liberibacter asiaticus” may be critical to survival of both endosymbionts, since phage-mediated lysis would likely breach the immunogenic threshold of the psyllid, invoking a systemic and nonspecific innate immune reaction.

## INTRODUCTION

Several Hemipteran insects transmit plant pathogens and have evolved cooperative associations with obligate, vertically transmitted bacterial endosymbionts owing to their strict reliance on a nutritionally restrictive diet. The Asian citrus psyllid *Diaphorina citri* Kuwayama (*Hemiptera*: *Liviidae*) harbors the stably associated intracellular gamma- and betaproteobacteria “*Candidatus* Carsonella ruddi” and “*Candidatus* Profftella armatura,” respectively, in specialized cells called bacteriocytes, which aggregate to form the bacteriome, a symbiotic organ within the psyllid body cavity (1). The obligate symbioses of “*Ca*. Profftella” (producing the defensive polyketide diaphorin) and “*Ca*. Carsonella” (producing required nutrients) with the psyllid host are considered ancient, and the two bacteria are mutually indispensable, with drastically reduced genomes of 0.5 and 0.17 Mb, respectively (2). The third psyllid endosymbiont, is an uncultured alphaproteobacterium of genus *Wolbachia* (order *Rickettsiales*). *Wolbachia* spp. are relatively widespread and estimated to infect >65% of insect species (3). So far, eight different supergroups of *Wolbachia* spp. have been recognized based on the 16S ribosomal sequences (4, 5), and some, but not all, are considered obligate endosymbionts. Diverse reproductive manipulations of host biology by *Wolbachia*, such as feminization, parthenogenesis, male killing, and cytoplasmic incompatibility are all aimed at enhancing transovarian passage of these bacteria to the next host generation (6).

*Diaphorina citri* has emerged as a devastating pest of citrus due to its role as a primary vector for the global epidemic of the highly destructive citrus tree decline disease called huanglongbing (HLB) or citrus “greening” (7). HLB is associated with three fastidious and uncultured alphaproteobacteria; “*Candidatus* Liberibacter asiaticus” in Asia and the Americas, “*Candidatus* Liberibacter africanus” in Africa, and “*Candidatus* Liberibacter americanus” present only in Brazil (8). In citrus, “*Ca*. Liberibacter asiaticus” is found only in phloem cells, from shoot tips to roots, causing severe disease symptoms after a surprisingly long incubation period (months to years) (9), and eventually killing infected trees. In contrast, “*Ca*. Liberibacter asiaticus” colonization in the psyllid host is systemic, spreading inter- and intracellularly across salivary glands, alimentary canal, Malpighian tubules, hemolymph, muscle and fat tissue, and ovaries, apparently with little or no manifestation of pathogenic effects (10). As a circulative endosymbiont that can replicate or accumulate to very high titers in some individuals, “*Ca*. Liberibacter asiaticus” is successful at evading the insect host immune system while traversing the midgut epithelium and basement membrane into the host hemocoel en route to the salivary tissues.

Most “*Ca*. Liberibacter asiaticus” “strains” examined thus far carry one or two prophages that are nearly identical and syntenic to SC1 and SC2 of strain UF506 (GenBank accession numbers NC_019549 and NC_019550, respectively) (11). The SC1 lytic cycle is marked by upregulation of several late genes, including a functionally lethal holin (SC1_gp110), and the cycle has been observed to be activated only when “*Ca*. Liberibacter asiaticus” is *in planta*, but not when infecting the psyllid host (12). Consistent with these observations is the fact that bacteriophage particles have been observed only in plant hosts, abundantly in the artificial host periwinkle (*Catharanthus roseus*) (11) and also in citrus (13). The holin gene (SC1_gp110) is the lead gene in the first operon of the late gene region of SC1 (11). The holin/late gene promoter drives strong and constitutive expression of β-glucuronidase (GUS) and green fluorescent protein (GFP) reporter genes in Liberibacter crescens, a culturable proxy for “*Ca*. Liberibacter asiaticus” (12, 14). *L*. *crescens* strain BT-1 was originally isolated from mountain papaya and is the only *Liberibacter* to be cultured thus far (15). There are no confirmed reports of pathogenicity or host colonization by strain BT-1, despite numerous inoculation attempts (14). Expression of the SC1 holin gene promoter fused with the GUS reporter in *L. crescens* was suppressed in a dose-dependent manner by crude aqueous extracts from psyllids, prompting us to hypothesize that prophage activation may limit “*Ca*. Liberibacter asiaticus” host range and culturability (12). We therefore sought to identify one (or more) ligand(s) in the psyllid extract capable of permeating *L. crescens* cells and binding and repressing the holin gene promoter.

## RESULTS

### SC1 lytic cycle genes are repressed in the psyllid host.

**FIG 1:**
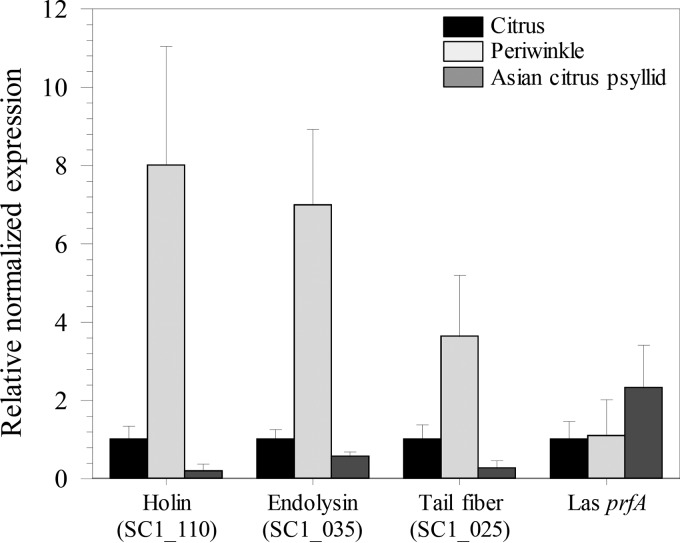
Relative expression of SC1 phage lytic cycle holin (SC1_gp110), endolysin (SC1_035), and tail fiber (SC1_gp025) genes and “*Ca*. Liberibacter asiaticus” (Las) *prfA* gene in psyllids, citrus, and periwinkle. The relative transcript abundance of each gene was normalized against expression levels of *gyrB* within each sample, and transcript abundance of psyllid and periwinkle samples was calibrated against expression levels in “*Ca*. Liberibacter asiaticus”-infected citrus. Values are averages (bars) plus standard errors of the means (error bars) (*n* = 3).

Comparative expression analyses of select “*Ca*. Liberibacter asiaticus” phage lytic cycle holin (SC1_gp110), endolysin (SC1_gp035), and tail fiber (SC1_gp025) genes were examined in comparison with “*Ca*. Liberibacter asiaticus” *prfA* in three different “*Ca*. Liberibacter asiaticus”-infected hosts: citrus, periwinkle, and psyllids. Transcript abundance in citrus was used as the baseline calibrator. The levels of expression of the holin, endolysin, and tail fiber were significantly lower in *D. citri* than the levels in citrus (*P* = 0.00012, 0.000615, and 0.004948, respectively) and dramatically lower than the levels in periwinkle (*P* = 0.000128, 0.000012, and 0.049213, respectively), corroborating both lack of a productive lytic cycle in psyllids and also the observed presence of phage particles *in planta*, most particularly in periwinkle (11). In contrast, “*Ca*. Liberibacter asiaticus” chromosomal gene *prfA* transcript levels were similar in both plant hosts and the insect host, demonstrating specific repression of bacteriophage late genes in *D. citri* (Fig. 1).

### The holin gene promoter is strongly constitutive in *L. crescens* but suppressed by exogenously applied psyllid extracts.

**FIG 2:**
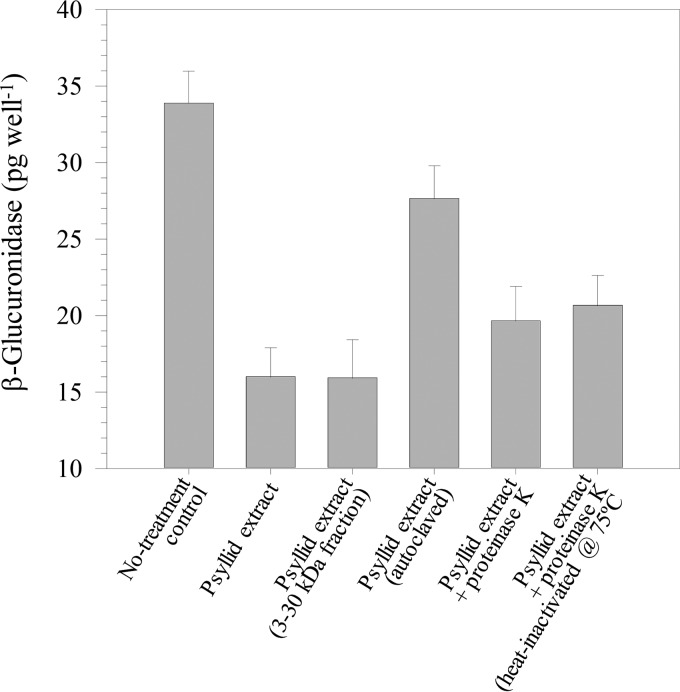
Effects of heat and proteinase K treatments on the inhibitory effect of crude aqueous psyllid extracts on fluorimetric GUS activity assays in *L*. *crescens* BT-1/pLF057 (*hol*::*uidA*) cells. For GUS activity inhibition treatments, 1-ml bacterial culture was incubated overnight with 50 µl psyllid extract, with or without a heat pretreatment (autoclaving at 121 lb/in^2^ for 20 min), 2.5 µl proteinase K solution (20 mg ml^−1^), or heat-inactivated proteinase K solution, as indicated. Proteinase K was heat inactivated by incubating at 75°C for 15 min.

The holin gene promoter was previously shown to be strongly and constitutively expressed in *L. crescens* strain BT-1 carrying pLF057 (*hol*::*uidA*); however, activity in BT-1 cells was suppressed in a dose-dependent manner by adding crude aqueous extracts from *D. citri* to the media (12). The inhibition of GUS reporter expression by exogenously supplied psyllid extracts was found to be both heat labile and susceptible to proteinase K inactivation, indicating involvement of a proteinaceous inhibitor; furthermore, the inhibitor appeared to be greater than 3 kDa but less than 30 kDa (Fig. 2). Together, these data led us to hypothesize that either *D. citri* and/or one of its bacterial endosymbionts produced one (or more) heat-sensitive proteinaceous inhibitors capable of regulating the SC1 lytic cycle activation through repression of the holin gene promoter.

### A holin promoter repressor protein is made by *Wolbachia*.

To identify the hypothetical protein able to suppress SC1 holin gene promoter activity, crude aqueous extracts of *D. citri* were size fractionated, and potential DNA-binding proteins from the 3- to 30-kDa fraction were affinity captured and subjected to liquid chromatography-tandem mass spectrometry (LC-MS/MS). These analyses resulted in peptide fingerprints representing 25 different proteins identified from multiple protein databases that most closely matched the MS/MS spectra of proteins captured in the sample. Two of these putative proteins were of endosymbiotic bacterial origin (*Wolbachia* and “*Ca*. Profftella armatura”), and the remaining proteins were from psyllids. Of the 25 proteins, the top hit was to a predicted hypothetical *Wolbachia* protein (WP_017531870) which yielded 22 unique spectra spanning 81% of the predicted 6.65-kDa protein. The remaining protein hits had significantly fewer unique spectra and lower coverage. The *Wolbachia* protein also satisfied the required search criteria: (i) small size (6.65 kDa); (ii) predicted DNA binding potential (DNAbinder) (16); (iii) predicted to be secreted (SecretomeP 2.0) (17); (iv) extracellular (PSLpred) (18). The 56-amino-acid (aa) putative repressor protein was encoded by WDIAC_RS0101550 annotated on Contig49.1 (GenBank accession number NZ_AMZJ01000058.1) from the draft metagenome assembly of *Wolbachia* strain wDi obtained from *D. citri*.

### The putative *Wolbachia* repressor protein binds “*Ca*. **Liberibacter** asiaticus” holin promoter DNA.

**FIG 3:**
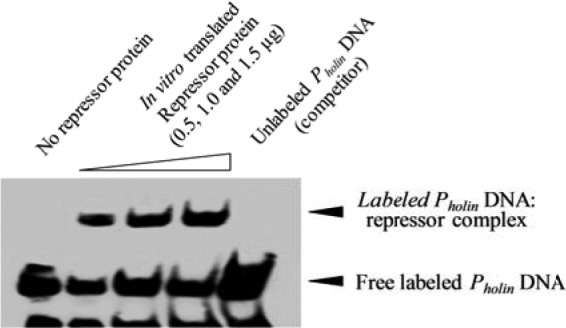
Holin promoter DNA-binding assay with *in vitro*-translated *Wolbachia* repressor protein WP_017531870. The leftmost lane contains labeled promoter DNA with no repressor protein. The middle three lanes contain increasing concentrations of repressor protein (indicated by the height of the triangle above the lane). The rightmost lane contains excess unlabeled promoter DNA with 1.0 µg repressor protein.

The WDIAC_RS0101550 gene was PCR amplified from *Wolbachia*-infected psyllids, sequenced, cloned in the pEXP5-CT expression vector, and used for *in vitro* cell-free protein synthesis. The *in vitro*-translated protein was size fractionated, concentrated, and used for examining its potential DNA binding activity. Electrophoretic mobility shift assay (EMSA) analysis of the interaction between biotinylated holin promoter DNA and the *in vitro*-translated *Wolbachia* wDi repressor protein revealed DNA binding activity (Fig. 3). Holin promoter DNA mobility was sharply decreased in the presence of the purified repressor, and the mobility shift was quenched by spiking the sample with unlabeled competitive DNA, confirming the specificity of DNA binding by this *Wolbachia* protein.

### The *Wolbachia* DNA-binding protein functionally represses the “*Ca*. **Liberibacter** asiaticus” holin promoter.

**FIG 4:**
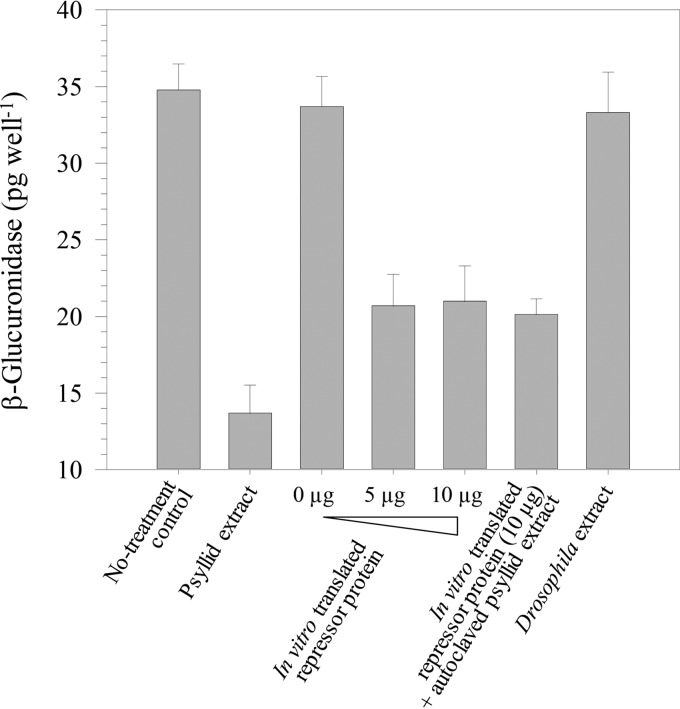
Inhibitory effect of *in vitro*-translated *Wolbachia* repressor protein on fluorimetric GUS activity assays in *L*. *crescens* BT-1/pLF057 (*hol*::*uidA*) cells. For GUS activity inhibition treatments, 1-ml bacterial cultures were incubated overnight with 50-µl psyllid extract, size fractionated, and incubated with concentrated (3 to 30 kDa) *in vitro*-translated protein (0, 5, and 10 µg as indicated) or crude aqueous *Drosophila* extract.

The *in vitro*-translated *Wolbachia* protein was added to liquid cultures of *L. crescens* BT-1 cells expressing the GUS reporter (*hol*::*uidA*) on pLF057, which replicates stably in BT-1. The repressor protein significantly inhibited GUS reporter activity driven by the holin promoter (Fig. 4). However, inhibition by the *in vitro*-expressed *Wolbachia* repressor was less than that observed using crude aqueous *D. citri* extracts. Addition of heat-inactivated psyllid extracts failed to enhance suppression of holin promoter activity mediated by the *Wolbachia* repressor protein (Fig. 4). Together, these observations indicate that complete suppression of the holin promoter requires either an additional interacting partner or posttranslational modification of the repressor by the psyllid.

### The *Wolbachia* DNA-binding protein was found only in the *Wolbachia* wDi genome from *D. citri*.

Comparative sequence analyses failed to uncover any homologs of the *Wolbachia* wDi repressor protein from any other *Wolbachia*-infected insects, including the complete genomic sequence of *Wolbachia* endosymbiotic strain wNo of Drosophila simulans (GenBank accession number NC_021084). As expected, crude aqueous extracts from *Wolbachia*-infected* Drosophila* failed to inhibit the GUS reporter in *L. crescens* cells (Fig. 4).

### The *Wolbachia* repressor protein is constitutively expressed in *D. citri*.

**FIG 5:**
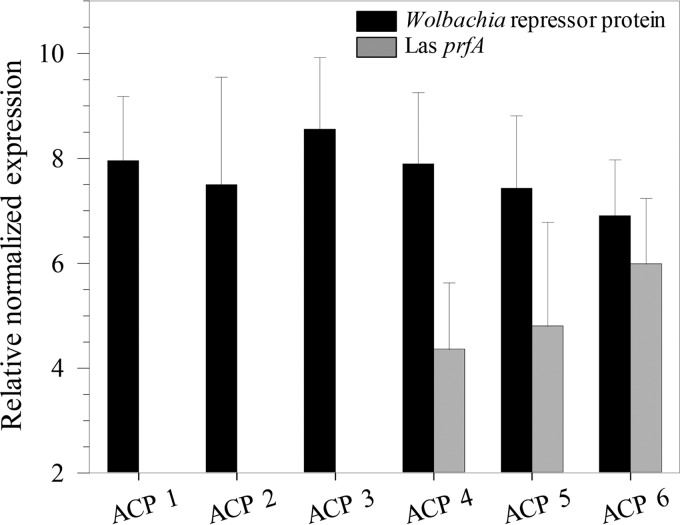
Expression of *Wolbachia*-encoded repressor protein in “*Ca*. Liberibacter asiaticus”-free healthy psyllids (ACP 1, 2, and 3) and “*Ca*. Liberibacter asiaticus”-infected psyllids (ACP 4, 5, and 6). Expression of *Wolbachia* repressor protein was normalized against the *Wolbachia wsp* gene in three independently extracted samples (10 insects each). “*Ca*. Liberibacter asiaticus” infection was verified using “*Ca*. Liberibacter asiaticus” (Las) *prfA* expression normalized against “*Ca*. Liberibacter asiaticus” *gyrB*. Values are averages (bars) plus standard errors of the means (error bars) (*n* = 6).

Quantitative reverse transcriptase PCR (RT-PCR) was carried out in “*Ca*. Liberibacter asiaticus”-infected and healthy (“*Ca*. Liberibacter asiaticus”-free) psyllids to determine whether the presence of “*Ca*. Liberibacter asiaticus” caused changes in the expression levels of the *Wolbachia* wDi gene-encoded repressor protein. Relative abundance of *Wolbachia* repressor protein (normalized against the expression of endogenous *Wolbachia* surface protein gene *wsp*) in six independent psyllid extracts (each with 10 insects) indicated that *Wolbachia* appeared to maintain constitutive expression of the repressor protein irrespective of the presence of “*Ca*. Liberibacter asiaticus” (Fig. 5).

## DISCUSSION

A productive lytic cycle is marked by transcriptional activation of phage late genes, including wall-degrading enzymes called endolysins and bacterial cell inner membrane-permeabilizing proteins called holins, allowing host cell lysis and phage particle egress (19). Functional holin and endolysin genes were confirmed carried on SC1 (SC1_gp110 and SC1_gp035, respectively), with the holin gene promoter encoding holin as the lead gene in the first operon of the late gene region (12). Consistent with lack of an observed “*Ca*. Liberibacter asiaticus” phage lytic cycle in psyllids and a single report of phage particles observed in citrus (13), comparative expression data revealed that transcription of the SC1 holin gene in “*Ca*. Liberibacter asiaticus” was significantly repressed in its insect host compared to citrus and dramatically suppressed compared to periwinkle (Fig. 1). To further investigate this repression, *L. crescens* was developed as a culturable proxy for uncultured “*Ca*. Liberibacter asiaticus,” allowing development of reporter gene assays using “*Ca*. Liberibacter asiaticus” promoter elements. The holin promoter was strongly and constitutively active in *L. crescens* and repressed in a dose-dependent manner by aqueous extracts of *D. citri* and plant hosts but to a lesser extent by extracts from plant hosts (12).

Preliminary EMSAs revealed binding of a factor(s) in the crude aqueous extracts from psyllids to the holin promoter DNA (not shown). Following affinity capture of proteins contained in the crude aqueous psyllid extracts, a predicted small protein encoded only by a gene carried by *Wolbachia* wDi found in psyllids was identified by LC-MS/MS. The gene encoding the 56-aa *Wolbachia* wDi protein was expressed *in vitro* and was functional in repressing a holin::GUS reporter in *L. crescens* when applied outside *L. crescens* cells (Fig. 4). The *Wolbachia* wDi repressor protein did not repress the GUS reporter in *L. crescens* in a dose-dependent manner and not to the level obtained by use of crude aqueous extracts from psyllids. Complete repression appeared to require (i) an enzymatic posttranslational modification in the psyllid, (ii) a cognate proteinaceous interacting partner of the repressor, or (iii) another independent proteinaceous repressor that is yet to be identified from psyllids and/or one of their endosymbionts.

All *D. citri* populations tested thus far are naturally infected with the *Wolbachia* strain wDi (supergroup B) (20). Given the ubiquitous prevalence of *Wolbachia* across all psyllids analyzed in Florida (21) and Brazil (22), it is highly likely that the inherent (primary and secondary) microbial communities of *D. citri* affect acquisition, establishment, and also transmission of “*Ca*. Liberibacter asiaticus.” However, the data on the population dynamics of “*Ca*. Liberibacter asiaticus” and other resident bacteria in *D. citri* remain largely descriptive. The titer of “*Ca*. Liberibacter asiaticus” was negatively correlated with the titers of the syncytium endosymbionts “*Ca*. Profftella” and “*Ca*. Carsonella” residing in the psyllid bacteriome (23). However, the *Wolbachia* titer in *D. citri* was greater in insects infected with “*Ca*. Liberibacter asiaticus” (23, 24).

In contrast to many insects, annotation of the *D. citri* draft genome revealed the complete absence of putative genes for the Imd pathway and known antimicrobial peptides (AMPs) in an attempt to accommodate symbioses with primary Gram-negative endosymbionts (25, 26). However, *D. citri* may be capable of detecting and resisting Gram-negative bacteria via the Toll and Janus kinase (JAK)/signal transducer and activator of transcription factor (STAT) pathways, and clearing any microbial infection through cellular immune responses (26, 27). The “*Ca*. Liberibacter asiaticus” genome has been annotated as encoding genes for several stereotypical pathogen-associated molecular patterns (PAMPs) including flagellin, diaminopimetic acid (DAP) in its peptidoglycan layer, and lipopolysaccharide (LPS) (28, 29). Several lines of evidence indicate a potential for recognition of “*Ca*. Liberibacter asiaticus” infection by the psyllid host immune system (30, 31). In contrast, *Wolbachia* bacteria are thought to neither activate nor repress the immune system in their native hosts (32) and appear to completely evade host immune recognition by sequestration within intracellular host-derived membranous vesicles (33). However, evasion by stealth does not safeguard *Wolbachia* against the risk of collateral damage as a consequence of other bacterial infections (34).

It is safe to presume that in an event involving activation of phage lytic cycle genes in “*Ca*. Liberibacter asiaticus”-infected psyllids, the resulting bacterial lysate would likely breach the immunogenic threshold and invoke an immune response as a consequence of release of additional immunogenic determinants that were previously sequestered within the intact cells. For example, phage particles and DNA, when delivered intracellularly in mice, altered the expression of innate immune genes (35), and transfection of bacterial DNA in *Drosophila* cells elicited an innate immune response (36). In addition, both purified LPS and LPS contained in outer membrane fragments are similarly more immunogenic than intact bacterial LPS (37). The *Wolbachia* repressor-encoding gene identified in this work from psyllids has not been found with any significant level of similarity at the protein level in any of the other sequenced *Wolbachia* genomes. We speculate that in response to the significant immunogenic potential posed in an event of SC1 lytic cycle activation in “*Ca*. Liberibacter asiaticus,” this repressor is a specific adaptation by *Wolbachia* in response to the presence of SC1, either originally in “*Ca*. Liberibacter asiaticus” or in another member of the psyllid endosymbiont community. Either way, it likely enhances survival prospects of both “*Ca*. Liberibacter asiaticus” and *Wolbachia* in psyllids.

“*Ca*. Liberibacter asiaticus” is clearly much better adapted to its psyllid host as an endosymbiont than to its citrus host as a pathogen. “*Ca*. Liberibacter asiaticus” crosses multiple cellular membranes of different organs (salivary glands, filter chamber, midgut, muscle tissue, and ovaries) in psyllids (38), lives in evident equilibrial symbioses with several other psyllid endosymbionts, and although it can cause localized apoptosis in psyllid midgut tissue, causes few overt disease symptoms (10). In contrast in citrus, “*Ca*. Liberibacter asiaticus” is limited to phloem and causes a debilitating citrus decline due to HLB, often resulting in tree death (7). Repression or loss of the SC1 phage lytic cycle may be critical for growth of “*Ca*. Liberibacter asiaticus” in psyllids.

One alternative to repression of the lytic cycle is selection for loss of either the phage or the lytic cycle genes of the phage. Defective prophage variants are thought to arise as a result of such selection pressure (39). Indeed, the majority (90.4%) of “*Ca*. Liberibacter asiaticus” strains surveyed in southern China carried only one of the SC1 or SC2 prophage variants, and the majority of those that carried one prophage variant were missing the lytic SC1 prophage (40). The SC2 prophage is notable for its lack of lytic cycle genes and a lysogenic conversion factor favorable for colonization of plants to encode an active peroxidase (14).

Plants and psyllids may or may not have an innate immune response adequate to both survive and clear *Liberibacter* infections, and clearly liberibacters have developed different strategies to suppress (14) or avoid triggering (29) such responses. Curing the psyllid of *Wolbachia* wDi carrying the repressor or mutating or deleting the repressor from the *Wolbachia* wDi genome might result in elimination and clearing of “*Ca*. Liberibacter asiaticus” or in lethality of the psyllid host. Since the holin reporter gene is constitutively active in *L. crescens* cells in the absence of a repressor, it is likely that the “*Ca*. Liberibacter asiaticus” holin and other lytic cycle genes would also be active in “*Ca*. Liberibacter asiaticus” as soon as axenic cultures are attempted without addition of host plant or psyllid extracts. The inability to culture “*Ca*. Liberibacter asiaticus” has been a significant obstacle to research progress in understanding and controlling this devastating citrus disease.

Although there are reports of (i) peptide-mediated quorum sensing in Gram-positive bacteria (41), (ii) antimicrobial lytic peptides that bind DNA (42), (iii) cysteine-rich plant peptides that can enter rhizobia and dramatically affect bacterial morphology (43), and (iv) homeodomain proteins (as long as 60 residues) being able to cross insect cellular membranes (44), to our knowledge, this is the first report of a protein repressor able to permeate a bacterial cell and directly affect gene expression. This also represents a new and unusual form of bacterial cross talk between genera that does not involve quorum sensing.

## MATERIALS AND METHODS

### Plant material and insect samples.

“*Ca*. Liberibacter asiaticus”-infected leaf samples were excised from the curated citrus (*Citrus paradisi*) and periwinkle (*Citrus roseus*) plants maintained in a quarantine greenhouse at the University of Florida, Gainesville. Healthy mixed sex adult psyllids (reared on “*Ca*. Liberibacter asiaticus”-free orange jasmine, *Murraya paniculata*) were provided by Eric Rohrig, Florida Department of Agriculture. “*Ca*. Liberibacter asiaticus”-infected insects (reared on “*Ca*. Liberibacter asiaticus”-infected sweet orange [*Citrus sinensis*]; approximately 80% infection density) were provided by David Hall, USDA, ARS. *Drosophila* cultures were obtained from the lab of Marta Wayne, Biology Department, University of Florida.

### Bacterial growth conditions and transformation.

The relevant characteristics and source and/or reference for the bacterial strains and plasmids used in this study are listed in Table 1. Escherichia coli was grown in Luria-Bertani (LB) medium at 37°C. *L. crescens* strain BT-1 was maintained in liquid BM7 medium containing 2 g alpha-ketoglutarate, 10 g *N*-(2-acetamido)-2-aminoethanesulfonic acid (ACES) buffer, and 3.75 g KOH in 550 ml water (pH 6.9) followed by addition of filter-sterilized 300 ml fetal bovine serum (HyClone Laboratories, Logan, UT, USA) and 300 ml modified Grace’s insect culture medium (TNM-FH; HyClone Laboratories), with moderate aeration at 150 rpm at 28°C. Electrocompetent BT-1 cells were prepared and transformed as previously described (12, 14). The following antibiotics were used as needed at the indicated concentrations: ampicillin (Amp), 100 µg/ml, kanamycin (Kn), 50 µg/ml, and gentamicin (Gm), 2 µg/ml.

**TABLE 1:** Bacterial strains and plasmids used in this study

Strain or plasmid	Relevant characteristics^a^	Source or reference
Strains		
E. coli Mach1-T1^R^	F^–^ φ80*lacZ*ΔM15 Δ*lacX74 hsdR*(r_K_^–^ m_K_^+^) Δ*recA1398 endA1 tonA*	Invitrogen
E. coli TOP10	F^–^ *mcrA* Δ(*mrr*-*hsdRMS*-*mcrBC*) φ80*lacZ*ΔM15 Δ*lacX74 recA1 araD139* Δ(*ara leu*)*7697 galU galK rpsL* (Str^r^) *endA1 nupG*	Invitrogen
* L. crescens*	Strain BT-1, originally isolated from mountain papaya	15
Plasmids		
pCR2.1-TOPO	3.9 kb; PCR cloning vector; Ap^r ^Kn^r^	Invitrogen
pEXP5-CT/TOPO	2.7 kb; PCR cloning vector; T7 promoter-based expression; C-terminal 6× His (HHHHHH-COOH); TEV recognition site (Glu-X-X-Tyr-X-Gln-Ser); Ap^r^	Invitrogen
pLF057	405-bp fragment of SC1_gp110 promoter region fused with promoterless *uidA* from pBI221 (*hol*::*uidA*) in pUFR071 with *lacZ* promoter deleted	12
pUFR071	9.4 kb; *repW* ColE1 Mob^+^ *lacZ*^+ ^Par^+^; Cm^r^ Gm^r^	12

a TEV, tobacco etch virus.

### Nucleic acid extractions from psyllid and plant samples.

DNA was extracted from plant leaf discs and from whole psyllids using the DNeasy plant minikit and blood and tissue kit, respectively (Qiagen, Valencia, CA, USA). The presence of “*Ca*. Liberibacter asiaticus” in the infected citrus samples was confirmed using conventional and nested PCR primer sets OI1/OI2c (45) and CG03F/CG05R (F stands for forward, and R stands for reverse) (46). For the extraction of total RNA, the midribs of PCR-confirmed “*Ca*. Liberibacter asiaticus”-infected leaves and psyllid samples (10 insects pooled together) were ground with a cold mortar and pestle in lysis buffer RLT (provided with Qiagen RNeasy plant minikit). RNA was extracted following the manufacturer’s protocol, diluted with nuclease-free water to 200 ng µl^−1^, and cleaned with Turbo DNA-free (DNase) kit (Ambion, Austin, TX, USA).

### Quantitative reverse transcriptase PCR.

The primer pairs used for the quantitative reverse transcriptase PCR (qRT-PCR) analyses are listed in Table 2. Reverse transcription reactions were performed using 1 µg RNA template (iScript Advanced cDNA synthesis kit; Bio-Rad, Hercules, CA, USA). Quantitative RT-PCR analyses were performed using a CFX96 Touch real-time PCR detection system (Bio-Rad, Hercules, CA, USA) by the method of Jain et al. (14). At least three biological replicates and four technical replicates were used with no-template and no-RT controls. Relative expression levels were calculated and normalized by the ΔΔ*C*_*T*_ method (47) and corrected for amplification efficiency. Data analyses and Student’s *t* tests (α = 0.05) were performed using the Bio-Rad CFX Manager Software package 3.0.

**TABLE 2:** Primers used in this study

Purpose or target and primer^a^	Sequence (5′→3′)	Reference
Las confirmation		
OI1	GCG CGT ATG CAA TAC GAG CGG C	41
OI2c	GCC TCG CGA CTT CGC AAC CCA T	41
CG03F	RGG GAA AGA TTT TAT TGG AG	42
CG05R	GAA AAT AYC ATC TCT GAT ATC GT	42
Quantitative RT-PCR		
wspDi_qF3	AGG GCT TTA CTC AAA ATT GG	25
wspDi_qR3	CAC CAA CGT ATG GAG TGA TAG G	25
wrpDi_qF	TGG ACA AAC TGA ATC CCA GTA TC	This study
wrpDi_qR	CAA ATC ATA CCC ACT GAT TCT TGA AC	This study
LasprfAF	TGT CTG AAT CGC CTT CTG TC	12
LasprfAR	GAT CAC CGA TGA CAG TAT GC	12
LasgyrBF	TTG AAC AAG CTG TAA TTT CTG G	12
LasgyrBR	ATC TGT TTG CCA ATT TAG AAG C	12
SC1_gp025F	AGC TAG ATC ATT GAC TCT TCC	12
SC1_gp025R	AAA GAT GTT GGT CGT AAA CTA G	12
SC1_gp035F	CGG TCT TCG CTA TGG ATT GA	This study
SC1_gp035R	TGG ATA AAG AGA CCG CTG ATG	This study
SC1_gp110F	TCG TAC ATG CAC CCC TGA TA	12
SC1_gp110R	AAG TGA GAC GCC AGG AAA GT	12
*Wolbachia* repressor protein		
wrpDiF	ATG CTA AAA CAC AAC GTT TTT GGT GAG A	This study
wrpDiR	TTA CTT GGT GCC GCC TAT TCT CCG T	This study
DNA affinity capture and EMSA		
HPromF	CGT ACG TGA CGC AAA TAA CAC TGG TGC	This study
HPromFBio	5Biosg-CGT ACG TGA CGC AAA TAA CAC TGG TGC	This study
HPromR	CCT AGG CCG ATA AAC TCC AAA AAA CGA G	This study
HPromRBio	5Biosg-CCT AGG CCG ATA AAC TCC AAA AAA CGA G	This study

a Las, “*Ca*. Liberibacter asiaticus.” In primer designations, F and R stand for forward and reverse, respectively, q stands for quantitative, Las stands for “*Ca*. Liberibacter asiaticus,” wrp stands for *Wolbachia* repressor protein, Prom stands for promoter, and Bio stands for biotinylated.

### Preparation of psyllid and *Drosophila* extracts.

Approximately 50 whole insects were pulverized to a fine powder under liquid N_2_ and resuspended in 1 ml deionized water. The resulting suspension was cleared by centrifugation twice at 3,220 × *g* for 20 min at 4°C, filter sterilized, and stored at −20°C. The psyllid extracts were heat inactivated by autoclaving (120 lb/in^2^, 20 min). For proteinase treatment, 50 µl psyllid extract was incubated with 2.5 µl (20 mg ml^−1^) proteinase K (New England Biolabs, Ipswich, MA) for 30 min, and the reaction was terminated by heating at 75°C for 15 min.

### β-Glucuronidase assay.

β-Glucuronidase (GUS) activity in *L. crescens* cells (carrying pLF057; *hol*::*uidA*) was examined using the fluorogenic substrate MUG (4-methylumbelliferyl-β-d-glucuronide; Sigma-Aldrich, St. Louis, MO). One-milliliter bacterial cultures were harvested at 2,013 × *g* for 15 min at 4°C, and the bacteria were resuspended in 30 µl GUS extraction buffer (50 mM Na_2_HPO_4 _[pH 7.0], 10 mM β-mercaptoethanol, 10 mM Na_2_EDTA [pH 8.0], and 0.1% Triton X-100) and incubated for 10 min at 37°C. Following the lysis reaction, 90 µl substrate solution (1 mM MUG in GUS extraction buffer) was added per 10 µl lysate and incubated at 37°C. Reaction aliquots (100 µl) were withdrawn at 10, 40, and 70 min, stopped with 50 µl of 0.2 M Na_2_CO_3_, and measured for fluorescence. The data were normalized against a standard curve prepared using the purified GUS enzyme (type IX-A from E. coli; Sigma-Aldrich). The data presented are mean values derived from two biological assays run in triplicate.

### DNA affinity capture assay.

A 535-bp fragment of the holin promoter region was PCR amplified using Accuprime *Taq* High Fidelity polymerase (Invitrogen, Carlsbad, CA), 2× Failsafe buffer D (Epicentre, Madison, WI) and the primer pair HPromF/HPromRBio (Table 2) and concentrated using the QIAquick PCR purification kit (Qiagen). Dynabeads (100 μl) (10 μg ml^−1^) (MyOne streptavidin C1; Invitrogen) were washed three times by gentle mixing in 1 ml of 1× binding and washing (BW) buffer (5 mM Tris-HCl [pH 7.5], 0.5 mM EDTA, 1 M NaCl). Washed beads were resuspended in 200 μl of 2× BW buffer and mixed with an equal volume of the 5′-biotinylated holin promoter DNA (~5 μg). The suspension was incubated by gentle inversion for 15 min and magnetically separated. The beads carrying the immobilized DNA were washed twice in 1× BW buffer. DNA-binding proteins were captured in 1× binding buffer (10 mM Tris, 50 mM KCl, 1 mM dithiothreitol [DTT] [pH 7.5]), 50 ng μl^−1^ poly(dI ⋅ dC), 5% glycerol, 5 mM MgCl_2_, 0.05% NP-40, 10 mM EDTA (pH 8.0), 10 μg protein (3- to 30-kDa-size-fractionated psyllid extract) in a final volume of 100 μl, and incubated for 20 min at room temperature. The beads were washed twice with 500 μl washing buffer (20 mM Tris [pH 8.0], 10 mM EDTA, 75 mM NaCl, 15% glycerol, 0.05% NP-40), and bound proteins were eluted by boiling for 5 min in 100 μl of 0.1% SDS. Affinity-captured proteins were resolved on precast 8 to 16% SDS-polyacrylamide gel (Mini-PROTEAN TGX; Bio-Rad). The protein bands were stained in Bio-Safe Coomassie blue stain (Bio-Rad), excised, and used for mass spectrometry.

Liquid chromatography-tandem mass spectrometry (LC-MS/MS) and protein search algorithm. The LC-MS analysis was carried out at the Interdisciplinary Center for Biotechnology Research (ICBR), University of Florida. In brief, the proteins were reduced, alkylated, and digested at 37°C overnight with trypsin (Promega, Madison, WI). After digestion, samples were injected into a capillary trap (LC Packings PepMap), desalted for 5 min with a flow rate of 3 μl min^−1^ of 0.1% (vol/vol) acetic acid, and subsequently loaded onto an LC Packing C_18_ Pep Map nanoflow high-performance liquid chromatography (HPLC) column. The elution gradient of the HPLC column started at 3% solvent A (0.1% [vol/vol] acetic acid, 3% [vol/vol] acetonitrile [ACN], and 96.9% [vol/vol] H_2_O) and 97% solvent B (0.1% [vol/vol] acetic acid, 96.9% [vol/vol] CAN, and 3% [vol/vol] H_2_O) and finished at 60% solvent A and 40% solvent B for 60 min for protein identification. LC-MS/MS analysis was carried out using an LTQ Orbitrap XL mass spectrometer (Thermo Fisher Scientific, West Palm Beach, FL) with an ion spray voltage of 2,200 V. Full MS scans were acquired from *m/z* ratios of 300 to 2,000 with a resolution of 60,000 in the Orbitrap mass spectrometer. All MS/MS samples were analyzed using Mascot (Matrix Science, Inc., London, UK; version 2.4.1). Mascot was set up to search the Diaphorina_citri_20151110 database (24,582 entries) assuming the digestion enzyme trypsin. Mascot was searched with a fragment ion mass tolerance of 0.01 Da and a parent ion tolerance of 10.0 ppm. Carbamidomethyl of cysteine was specified in Mascot as a fixed modification. N-terminal modification Gln→pyro-Glu, deamidation of asparagine and glutamine, and oxidation of methionine were specified in Mascot as variable modifications.

Scaffold (version Scaffold_4.4.1.1; Proteome Software Inc., Portland, OR) was used to validate MS/MS-based peptide and protein identifications. Peptide identifications were established with >80.0% probability by the Peptide Prophet algorithm (48) with Scaffold delta-mass correction. Protein identifications were accepted if they could be established at greater than 80.0% probability and contained at least two identified peptides. Protein probabilities were assigned by the Protein Prophet algorithm (49).

### Electrophoretic mobility shift assay.

Biotinylated holin promoter probe and analogous promoter fragment were PCR amplified using biotinylated (HPromFBio/HPromRBio) or standard primers (HPromF/HPromR) (Integrated DNA Technologies, Inc., Coralville, IA) (Table 2). Electrophoretic mobility shift assays (EMSAs) were carried out with PCR-purified promoter fragments using the LightShift chemiluminescence EMSA kit (Thermo Scientific) following the manufacturer’s protocol. Binding reactions were carried out in a cocktail containing 1× binding buffer (10 mM Tris, 50 mM KCl, 1 mM DTT, pH 7.5), 50 ng µl^−1^ poly(dI ⋅ dC), 2.5% glycerol, 0.05% NP-40, 10 mM EDTA (pH 8.0), 5 mM MgCl_2_, 50 mM KCl, 20 mM EDTA, 1 µg protein extract, and approximately 20 fmol biotin end-labeled promoter probes. Control reactions also included approximately 4 pmol unlabeled competitive binding promoter DNA. Binding reactions were resolved at 100 V on 6% native polyacrylamide gels in 0.5× Tris-borate-EDTA (TBE) and transferred to nylon membranes for 1 h at 100 V. After UV cross-linking, biotin-labeled DNA was detected using the chemiluminescent nucleic acid detection module (Thermo Scientific) following the manufacturer’s protocol.

### Cloning and expression of *Wolbachia* repressor protein.

The gene encoding the *Wolbachia* repressor protein was PCR amplified using primers wrpDiF/wrpDiR (Table 2), directly cloned in the pEXP-5-CT/TOPO TA expression vector and transformed into E. coli TOP10 cells (Invitrogen) according to the manufacturer’s recommendations. Following sequence verification, purified plasmid DNA was used for *in vitro* translation (PURExpress *in vitro* protein synthesis kit; NEB), and the resulting protein was concentrated using Amicon Ultra Centrifugal filter columns (molecular weight of 3,000 [3K] and 30K) (EMD Millipore, Millipore Corp., Billerica, MA). Total protein was quantified by Bradford protein assay using bovine serum albumin (BSA) as the standard (Bio-Rad).
